# Photonic passbands induced by optical fractal effect in Cantor dielectric multilayers

**DOI:** 10.1371/journal.pone.0268908

**Published:** 2022-08-02

**Authors:** Jianxia Liu, Jing Shen, Dong Zhao, Pu Zhang

**Affiliations:** 1 School of Electrical and Information Engineering, Hubei University of Science and Technology, Xianning, China; 2 Research Center for the Development of Rural Education and Culture, Key Research Base of Humanities and Social Sciences in Hubei Province, Hubei University of Science and Technology, Xianning, China; Information Technology University, PAKISTAN

## Abstract

We investigate the splitting and incorporation of optical fractal states in one-dimensional photonic quasi-crystals. The aperiodic crystals which are composed of two different dielectrics submit to Cantor sequence. Defects in Cantor crystals can greatly enhance the localization of electric field, which induces the optical fractal effect. The number of optical fractal states increases exponentially with the generation number of Cantor sequence. Moreover, the optical fractal characteristics depend on the incident angle of light, of which the fractal states may split/incorporate by modulating the value of incident angle. This study could be utilized for band-pass filters and reflectors.

## 1. Introduction

Fractal concept was first introduced by Mandelbrot in 1967 [1]. Fractal is an universal phenomenon in nature and has covered a wide range of the modern science from geography to nonlinear dynamics [2–4]. A fractal can be constructed based on the incorporation of identical motifs that repeat on different size scales. In optics issue, fractal structures can be contributed by three types, namely perfectly periodic media, absolutely random media and quasi-periodic media, respectively [5–7]. Compared with the periodic photonic crystals, quasi-periodic photonic crystals have displayed many unique features and have the particular interest for scientists [8–11].

Cantor sets of dielectric isotropic layers are the typical model fractal structures. Cantor-like dielectric superlattices (DSLs) have been studied widely on the structural and physical properties since Cantor sets were defined by G. Cantor in 1883 [12]. In contrast with Thue-Morse- and Fibonacci- like superlattices, the unit cell thickness of Cantor type DSLs increases with the sequence step [13–15]. Many excellent features and behaviors were concerned by the scientists around the word in the past twenty years. Such as, the self-similarity behavior of the reflectance and transmission spectra of Cantor type DSLs [16], the effective index and third order nonlinear susceptibilities of Cantor structures [17], the scaling properties of optical Cantor filters [18], the second harmonic generation and the third harmonic generation of Cantor type DSLs [19], the photonic bandgap behavior and the optical windows presence of one-dimensional triadic Cantor quasi-periodic structures [20], the tunable multichannel filter property based on the use of periodic Cantor dielectric multilayers [21], the second harmonic generation in Cantor like metamaterial photonic superlattices [22], the unidirectional absorption properties of the defective asymmetric Cantor photonic crystals [23], the self-similarity feature of complex quasi-periodic Fibonacci and Cantor photonic crystals [24].

In this work, the studies only focus on the Cantor dielectric multilayers that are quasi-periodic (or aperiodic) photonic crystals composed of two different dielectrics Si and SiO_2_. The discussions are summarized as follows: (a) Transmission spectra and the distributions of the electrical field are analyzed using one-dimensional transfer matrix method. (b) Optical fractal effect is analyzed and discussed. The formula to compute the number of optical fractal states is established. The results show that it is matched very well between the formula calculation and the numerical simulation. (c) Optical fractal effect is analyzed with the change of incident angle of light. We find that the optical fractal states may split/incorporate by modulating the value of incident angle. The pass-bands are produced with the incorporation of the optical fractal states. It is a candidate to be applied in band-pass filters.

## 2. Cantor multilayers

**Fig 1:**
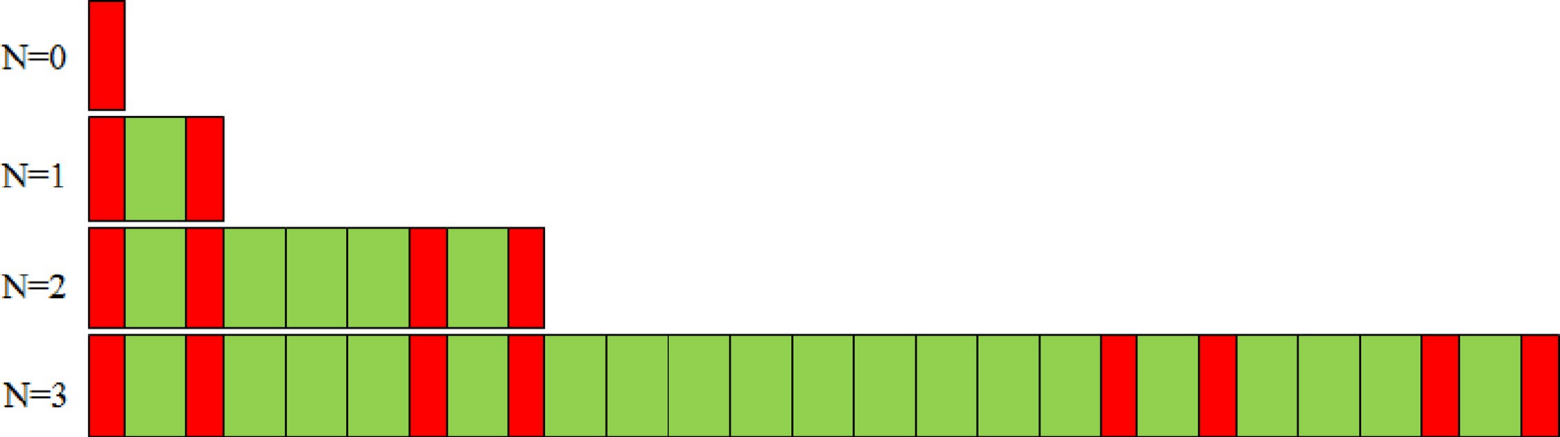
Diagram of the Cantor dielectric multilayers for *N* = 0, 1, 2, 3, respectively. The symbol of *N* is the generation number of the Cantor sequence.

In this study, an one-dimensional photonic quasi-crystal is proposed. It is an aperiodic crystal which is composed of two different dielectrics and submits to Cantor sequence law [25]. Each of the Cantor dielectric multilayers is characterized by a fundamental parameter *N*, *viz*. the generation number *N*, where *N* is an integer of 0, 1, 2, 3 …… in turn. There is only one layer for *N =* 0. With the increase of the generation number *N*, the number of the crystal layers will increase rapidly with 3^*N*^ law. For instance, the number of the crystal layers is 3, 9 and 27 for the generation number *N* = 1, 2 and 3, respectively. Fig 1 gives the diagram of the Cantor dielectric multilayers for different generation number *N*. The red layers represent the higher index dielectric Si and the green layers represent lower index dielectric SiO_2_. The refractive indices of the two dielectrics Si and SiO_2_ are *n*_*a*_ = 3.53 and *n*_*b*_ = 1.46, respectively [26, 27]. The thicknesses of dielectric slabs are 1/4 optical wavelengths, *viz*. *d*_*a*_ = λ_0_/4/*n*_*a*_ = 0.1098 μm (denoted in red) and *d*_*b*_ = λ_0_/4/*n*_*b*_ = 0.2654 μm (denoted in green), where λ_0_ = 1.55 μm is the central wavelength. The substrate surrounding of the aperiodic system is air and its refractive index is set as 1.

**Fig 2:**
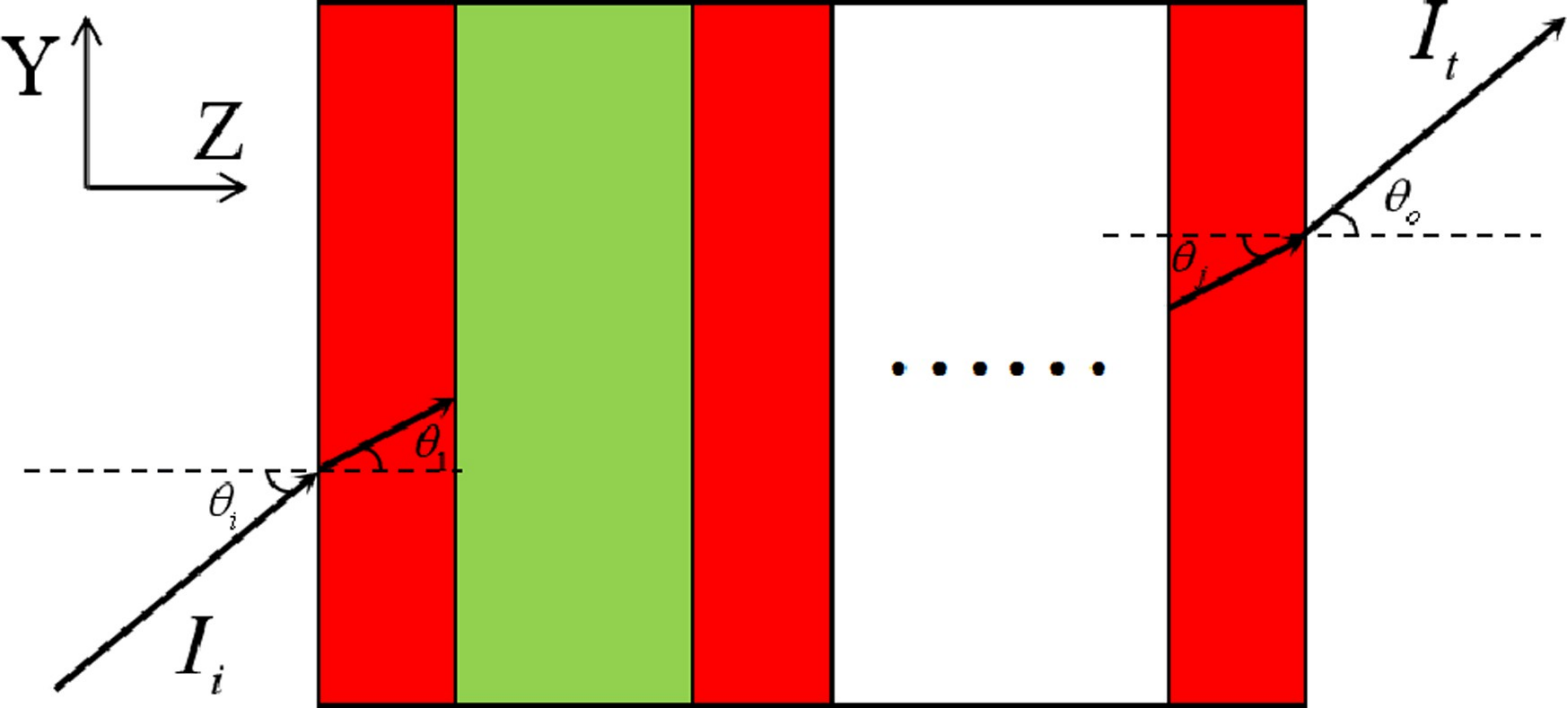
Light path diagram of optical wave transmitting in the Cantor dielectric multilayers.

The transfer matrix method [28] is used to investigate the transmission spectra of light in the Cantor dielectric multilayers. The path diagram of the optical wave transmitting in the Cantor dielectric multilayers is shown in Fig 2. The alphabet *θ*_*i*_ is the incident angle of light from the air to the multilayer medium and *θ*_*o*_ is the output angle of light from the multilayers to the external environment. The horizontal direction to the right is the positive direction of the Z-axis and the vertical up direction is the positive direction of the Y-axis. The transfer matrix of each layer is written in the following form of

$$T_{j}=\left[\begin{array}{cc} \cos\;\delta_{j} & \frac{i}{\eta_{j}}\;\sin\;\delta_{j}\\ i\eta_{j}\;\sin\;\delta_{j} & \cos\;\delta_{j} \end{array}\right].$$ (1)

The global transfer matrix of the Cantor dielectric multilayers is obtained by

$$\begin{array}{c} T_{global}=T_{1}T_{2}\cdot \cdot \cdot \cdot \cdot \cdot T_{j}\\ =\left[\begin{array}{cc} \cos\;\delta_{1} & \frac{i}{\eta_{1}}\;\sin\;\delta_{1}\\ i\eta_{1}\;\sin\;\delta_{1} & \cos\;\delta_{1} \end{array}\right]\left[\begin{array}{cc} \cos\;\delta_{2} & \frac{i}{\eta_{2}}\;\sin\;\delta_{2}\\ i\eta_{2}\;\sin\;\delta_{2} & \cos\;\delta_{2} \end{array}\right]\cdot \cdot \cdot \cdot \cdot \cdot \left[\begin{array}{cc} \cos\;\delta_{j} & \frac{i}{\eta_{j}}\;\sin\;\delta_{j}\\ i\eta_{j}\;\sin\;\delta_{j} & \cos\;\delta_{j} \end{array}\right] \end{array}$$

$$\text{=}\left[\begin{array}{cc} A & B\\ C & D \end{array}\right],$$ (2)

where A, B, C and D are the matrix elements depending on the parameters of the Cantor dielectric multilayers, *δ*_*j*_ is the phase shift of the light in the (*j*–1)- and the *j*-th layer. The phase shift of light *δ*_*j*_ is calculated using the following formula

$$\delta_{j}=\frac{2\pi }{\lambda }n_{j}d_{j}\;\cos\;\theta_{j},$$ (3)

where *η*_*j*_ is the effective optical admittance of the *j*-th layer and is calculated by

$$\eta_{j}=\eta_{0}n_{j}\;\cos\;\theta_{j},$$ (4)

where $\eta_{0}=\sqrt{\frac{\varepsilon_{0}}{\mu_{0}}}$ is the optical impedance admittance in vacuum, *n*_*j*_ is the refractive index of the *j*-th layer and *d*_*j*_ = λ_0_/4*n*_*j*_ is the *j*-th layer thickness, *λ*_0_ is the reference wavelength of light and *λ* is the working wavelength of light.

The transmission coefficient of electric filed is calculated by

$$t=\frac{2\eta_{j}}{A\eta_{j}+B\eta_{j}\eta_{0}+C+D\eta_{0}}.$$ (5)

The intensity of the output light is obtained by

$$I_{o}=I_{i}{\left|t\right|}^{2}=I_{i}T,$$ (6)

where *I*_*i*_ is the input intensity of light and *T* is named as the transmittance of optical intensity.

## 3. Optical fractal effect

Optical fractal is a typical effect in the quasi-periodicity photonic crystals. The resonant states expand exponentially as the generation number *N* of photonic crystals increases. We here demonstrate the transmission spectra of lights in the Cantor dielectric multilayers for different values of *N*.

**Fig 3:**
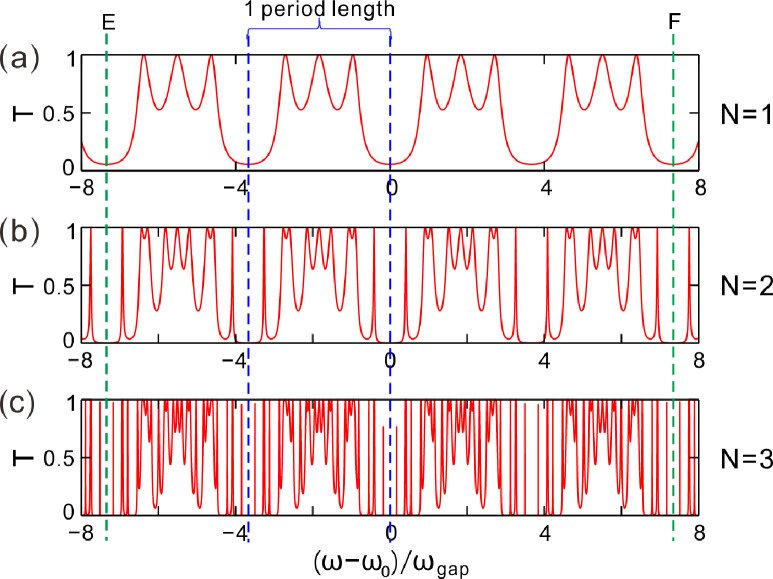
(**a-c**) Transmittance varying with the frequencies of the incident light wave for the generation number *N* = 1, 2 and 3, respectively. Here, incident angle of light *θ*_*i*_ = 0°.

For a transverse magnetic (TM) wave, as it impinges on the Cantor photonic crystal from the left, Fig 3A describes the transmission spectrum of light for *N* = 1. The horizontal coordinate (ω – ω_0_)/ω_gap_ is the normalized frequency of the incidence light waves, where ω = 2πc/λ, ω_0_ = 2πc/λ_0_ and ω_gap_ = 4ω_0_arcsin│[Re(*n*_*H*_) − Re(*n*_*L*_)]/[Re(*n*_*H*_) + Re(*n*_*L*_)]│^2^/π are respectively called as the incident angular frequency of light, the central angular frequency and photonic bandgap. c is the vacuum velocity of light. We here borrow the concept of photonic bandgap in periodic photonic crystals and give four spectral periods for each generation number *N*. One period length has been noted in the area between two blue dotted lines. Four periods have been noted in the area between green dotted lines E and F. There are 3 resonance peaks in one spectral period and 12 resonance peaks in four spectral periods for *N* = 1.

For *N* = 2, the resonant states increase to 9 in a period unit of transmission spectrum. The total quantity is as high as 36 in four period units as shown in Fig 3B. In the same way, 27 resonance peaks can be counted in one spectral period in Fig 3C and the number of the valid resonance peaks in four spectral periods is 108. Moreover, some peaks are produced by splitting and some others are new generating. Obviously, the optical fractal effect of the Cantor dielectric multilayers is observed with the increase of the generation number *N*.

**Fig 4:**
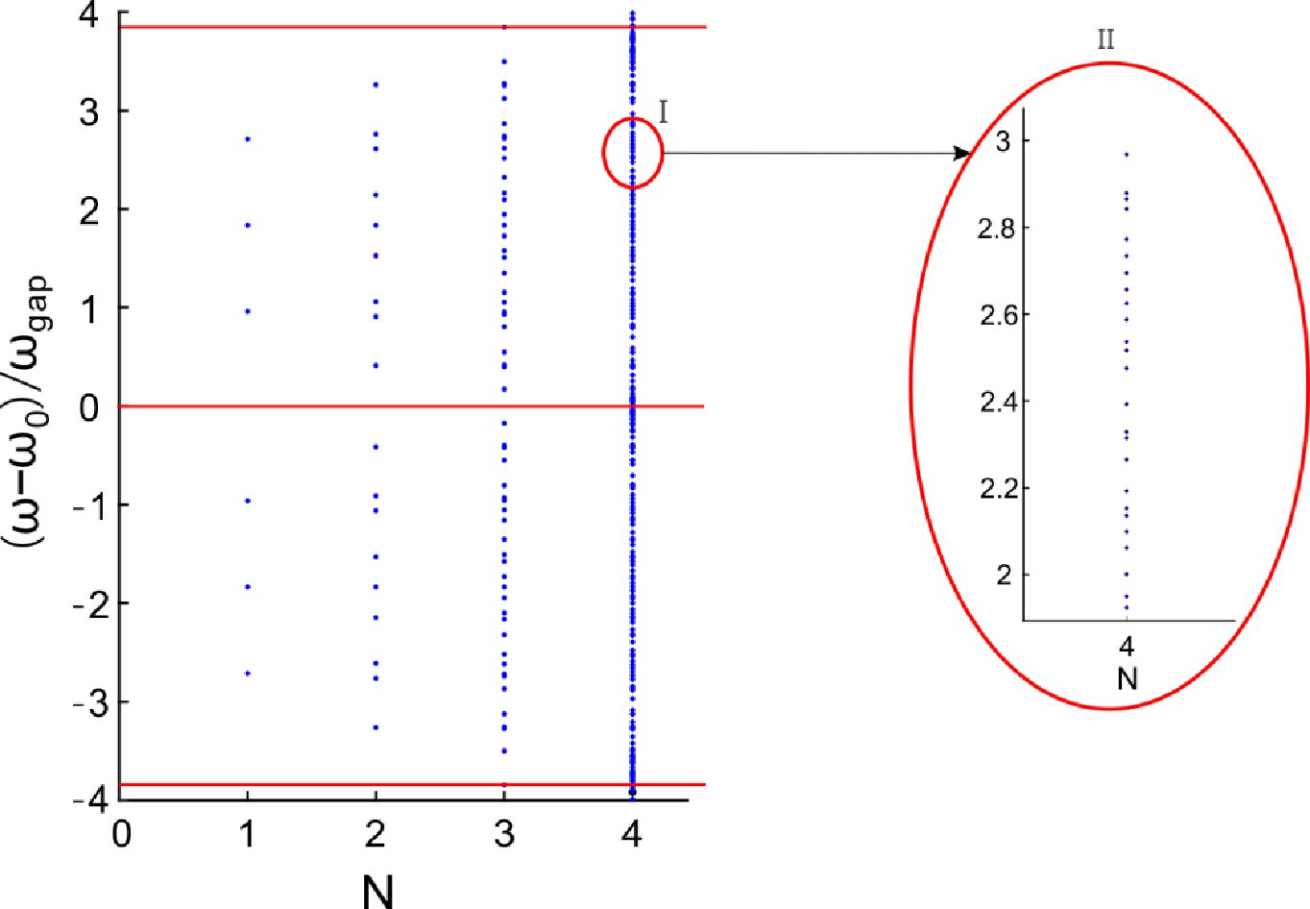
Frequencies of resonance peaks versus the generation number *N =* 1, 2, 3 and 4, respectively.

Resonance frequencies versus different generation number *N* for two spectral periods are given in Fig 4. The number of the resonance peaks for *N* = 1, 2 and 3 is counted respectively. The number of the resonance peaks increases exponentially with the increase of the generation number *N*. Because each resonant peak represents an optical fractal state, the optical fractal states increases proportionally with *N* as well. According to Fig 4, the number of the optical fractal states is counted for the generation number *N* = 1, 2 and 3, respectively. Because the number of the optical fractal states is large for the generation number *N* = 4, it is not clear to be demonstrated. For showing obviously the number of the optical fractal states, the area I is amplified and the details are displayed in subgraph II.

According to the above discussion, the optical fractal effect in the Cantor dielectric multilayers is expressed as sequential splitting. Namely, a spectral line is splitting into multiplets. The reason for the sequential splitting can be understood from the point of view of self-similarity of the Cantor dielectric multilayers regarded in terms of coupled cavities. At the same time, some new spectral lines also generate in forbidden bands. The number of the sequential splitting and the new generated spectral lines are respectively expressed by

$$P_{new}=\frac{G-1}{2},$$ (7)

$$P_{splitting}=\frac{G+1}{2}.$$ (8)

The total number of the spectral lines is computed by

$$P_{total}=G^{N},$$ (9)

where *G* is the generator and *N* is the generation number. In this work, the generator *G* is set to 3. According to the Eq (9), the total number of the splitting spectral lines is 3, 9, 27, and 81 for the generator *G =* 3 and the generation number *N* = 1, 2, 3 and 4, respectively. The quantitative relationship between the total spectral lines and the generation number *N* has been showed in Table 1. Compared with the features in Figs 3 and 4, it is matched very well between the theoretical calculation and the numerical simulation in Table 1.

**Table 1:** Quantitative relationship between the total spectral lines and the generation number *N*.

*N*	*P*_total_
1	3
2	9
3	27
4	81

**Fig 5:**
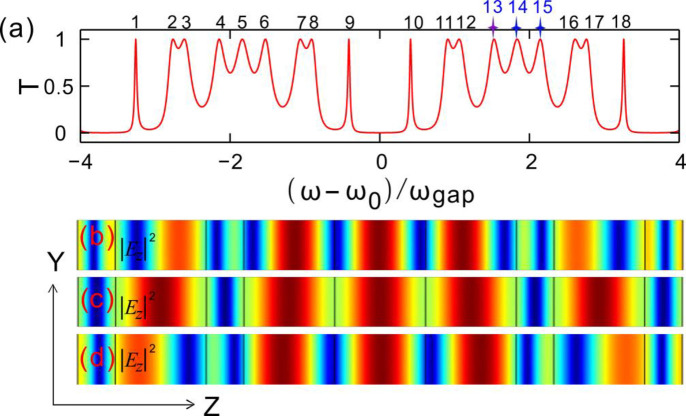
(**a**) Transmittance varying with the frequencies of incident light wave for the generation number *N* = 2. (**b-d**) Electric field distributions of the 13-th, 14-th and 15-th optical fractal state, respectively. The incident angle of light is *θ*_*i*_ = 0°.

For observing the electric field distributions of optical fractal states in the Cantor dielectric multilayers, two spectral periods of transmission for the generation number *N* = 2 are displayed in Fig 5A. There are 18 resonance peaks marked, *viz*. 18 optical fractal states. Three optical fractal states are arbitrarily selected, *viz*. the 13-th, 14-th, and 15-th peak, and their electric field distributions are simulated as well. The corresponding wavelengths are the values of *λ*_13,14,15_ = 0.7149, 0.7759 and 0.8461 μm, respectively.

The finite difference time domain (FDTD) is taken to simulate the electric field distributions. Fig 5B gives the electric field distribution for *λ*_1_ = 0.7149 μm. The mode field power is mainly distributed in the low index defects and the intensity decays from the center to both the left and right sides exponentially. The electric fields are stronger in the three center defects than these of the two terminal defects in the dielectric multilayers. At the same time, the electric field distribution shows a good central symmetry. Here the optical loss in the dielectrics is not considered since the loss of dielectrics is low and it does not impress optical fractal effect severely. The transmission peaks are almost coincident for the case containing loss in dielectrics and the case without loss. The corresponding electric field distribution of *λ*_1_ is more concentrated than that of *λ*_*2*_ and *λ*_3_.

Fig 5C gives the electric field distribution for *λ*_2_ = 0.7759 μm. The mode field is distributed in the low index defects and decays from the center to both the left and right sides. The electric field distribution shows a good central symmetry as well. However, the corresponding electric field distribution of *λ*_2_ is quite different from that of *λ*_1_. The maximum power of electric field locates in the three middle defects and the two terminal defects of the dielectric multilayers. There is not an almost forbidden band in the three middle defects for the electric field transmission. Therefore, it is easy to produce a passband with the central wavelength of *λ*_2_.

Fig 5D gives the electric field distribution for *λ*_3_ = 0.8461 μm. The mode field power is mainly distributed in the low index defects and decays from the center to both the left and right sides. The electric fields are stronger in the three center defects than that of the two terminal defects of the dielectric multilayers. The electric field distribution shows a good center symmetry as well. The distribution is similar with that of *λ*_1_. However, comparing with the electric field distribution of *λ*_1_, the band width of either pass-band or forbidden band is wider than that of *λ*_1_. In addition, the electric field intensity reverses between the wavelength *λ*_1_ and the wavelength *λ*_2_.

Defects in photonic crystals can greatly localize the electric field [29]. Defects in Cantor crystals can tremendously enhance the localization of electric field as well, which results in the optical fractal effect in aperiodic structures. The periodic photonic crystals support the photonic bandgap, while for the defective photonic crystals, the defect resonance modes, *viz*. transmitted modes, arise in the bandgap. The Cantor photonic crystals are aperiodic and many defects exist in the systems, so it approves a series of resonant transmitted states in the spectra. The resonant transmitted states split as the generation number increases. Consequently, the defect resonant modes in aperiodic photonic crystals are called the optical fractal states. The passband results from the incorporation of optical fractal states by modulating the incident angle. Therefore, the optical fractal effect is absent in the periodic photonic crystals. Otherwise, there are passbands on both sides of the bandgap as well, and the transmittance in this mentioned passband fluctuates as the frequency of light wave changes, of which the corresponding transmission peak is the standing wave resonance.

## 4. Fractal states incorporation and splitting

**Fig 6:**
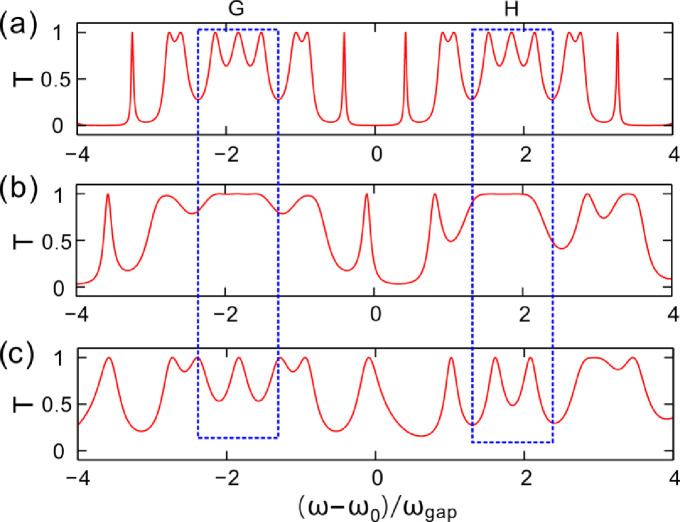
(**a-c**) Transmittance varying with the frequency of the incident light wave for the incident angle of 0°, 65°and 80°, respectively. Here, the generation number is *N* = 2.

The spectral properties of the dielectric multilayers are analyzed through changing the incident angle of light. Two spectral periods on different incident angles of light are shown in Fig 6. The generation number is *N* = 2. Fig 6A shows the spectrum for the incident angle of light *θ*_*i*_ = 0°. The spectral distribution is the same with that in Fig 5A and there are 18 optical fractal states in the transmission spectrum of light.

Fig 6B shows the spectrum for the incident angle of light *θ*_*i*_ = 65°. The spectrum is quite different from that of the incident angle *θ*_*i*_ = 0°. The number of the optical fractal states changes from 18 to 9. The number has been reduced by a half. The two pass bands appear and are marked in two blue rectangles G and H. In Fig 6A and 6B, the incorporation of the optical fractal states is resulted in the Cantor dielectric multilayers with the change of the incident angle of light from 0° to 65°. The 4-th, the 5-th and the 6-th optical fractal state in Fig 6A incorporate into the third optical fractal state in Fig 6B (Denoting the optical fractal states from the left to right). The 13-th, the 14-th and the 15-th optical fractal state in Fig 6A incorporate into the seventh optical fractal state in Fig 6B.

For the incident angle of light *θ*_*i*_ = 80°, the spectrum of light is showed in Fig 6C. The change of the spectrum is obvious. Comparing with Fig 6B and 6C shows that some optical fractal states split and two pass bands disappear. The band optical fractal states confined in the areas of the blue rectangle G in Fig 6B do not split as the incident angle increases, but it gradually forms an optical fractal state with a narrow bandwidth. In addition, the pass band of transmission spectrum in the right rectangular H in Fig 6B splitts into two optical fractal states as the incident angle is *θ*_*i*_ = 80°.

The Cantor photonic crystals investigated here are passive, of which the material loss is weak and has been ignored in the whole simulation process to highlight the optical fractal effect, so the systems are not non-Hermitian. Especially, the Cantor photonic crystals are aperiodic and the defects increase with the increase of the generation number of sequence, which demonstrates more resonant states induced in the transmission spectra and the states split by increasing the generation number, *viz*. optical fractal effect. Otherwise, the resonance of each fractal state decreases and the width of resonant peak widens as the incident angle increases, which results in two adjacent resonant peaks overlapping each other and connecting together to form a passband, while for the non-Hermitian system, the eigenvectors and eigenvalues coalesce at the exceptional point (EP) [30]. Therefore, the merging of optical fractal states in aperiodic structures differs from the coalescence of the eigenvectors and eigenvalues at EP in non-Hermitian systems. Otherwise, non-Hermitian systems have been intensively investigated to be utilized for electric field localization, solitons, topological edge states, topological insulators and chiral mode transfers [31–38].

Therefore, the optical fractal states incorporate as the increase of the incident angle from the incident angel *θ*_*i*_ = 0°. The wide bandpass is obtained as the incident angle of light increases to *θ*_*i*_ = 65°. As the incident angle of light continues to increase to *θ*_*i*_ = 80°, the splitting of the optical fractal states is observed again. However, the splitting of the optical fractal states cannot be recovered into the beginning profile. Some new optical fractal states are formed. Through selecting suited incident angle of light, the optical fractal states of the spectra are modulated. The characteristics could be utilized to design the band-pass filters and reflectors.

**Fig 7:**
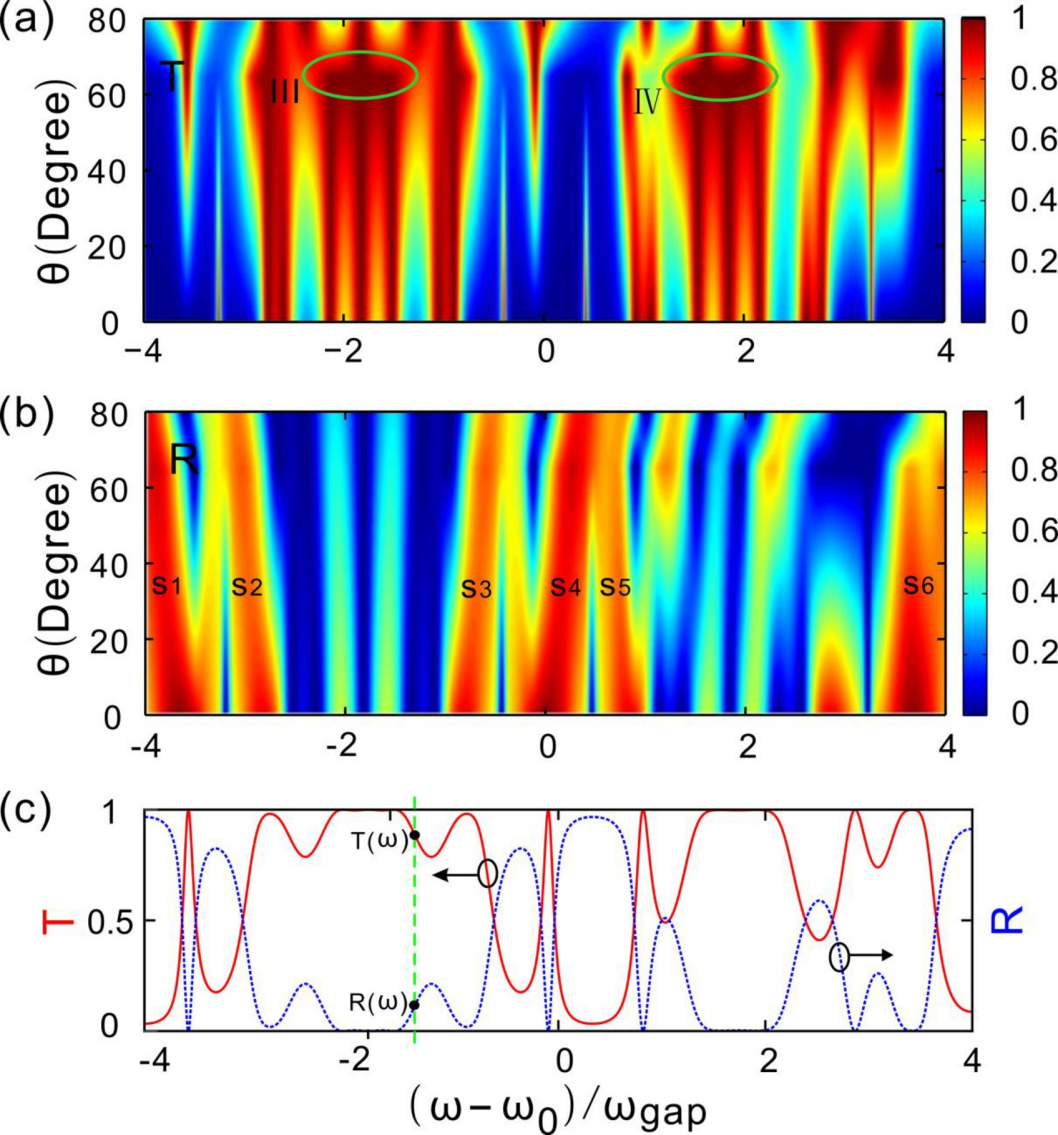
(**a, b**) Transmittance and reflectance versus the incident angle of light and the normalized frequency, respectively. (**c**) Transmission spectrum and the reflectance spectra for incident angle *θ*_*i*_ = 65°.

For presenting more clearly the incorporation and the splitting of the optical fractal states. The transmittance and the reflectance versus the incident angle of light and the normalized frequency are given. Fig 7A presents the transmittance as the change of the incident angle of light and the normalized frequencies. The incorporation of the optical fractal states is observed obviously as the incident angle of light increases. Two wide passbands appear in the areas marked in two green ellipses Ⅲ and Ⅳ, respectively. The two wide passbands produce as the incident angle of light is *θ*_*i*_ = 65°. The widths of the two passbands become narrower as one keeps increasing the incident angle of light. The splitting appears for the incident angle of light *θ*_*i*_ = 80°.

Fig 7B presents the reflectance as the incident angle of light and the normalized frequency increases. The intensity distributions are the opposite of that of the transmittance in Fig 7A. It is well known that the maximum of transmittance corresponds to the minimum of reflectance. Obviously, it is matched very well between the simulation results and the theory. Six good reflective bands are observed and are marked as S1, S2, S3, S4, S5, and S6, respectively. Using the properties of the reflectance of the Cantor dielectric multilayers, the multi-wavelengths reflectors can be designed as well.

The Cantor photonic crystals here are passive, which dominates the sum of transmittance and reflectance *T* + *R* ≤ 1. To demonstrate this property more clearly, we gives the transmission and reflection spectra changing with the normalized frequency for a fixed incident angles *θ*_*i*_ = 65° as shown in Fig 7C. One can see that, for each given incident frequency of light wave, the value satisfies the formula *T*(*ω*) + *R*(*ω*) ≤ 1. Changing the incident angle, the above expression is true as well.

In fact, both transverse magnetic and transverse electric polarized waves can be used to achieve optical fractal effect and photonic passbands in Cantor photonic crystals here. Avoiding repetition, we here only gives the spectra of light for the transverse magnetic waves.

## 5. Conclusions

In conclusion, the splitting and incorporation of optical fractal states in one-dimensional photonic quasi-crystals have been studied. The aperiodic crystals which are composed of two different dielectrics submit to the Cantor sequence law. Defects located in the Cantor multilayers can restrict greatly the power of the electric fields, which approves to induce an optical fractal effect. Scalability and sequential splitting of the spectra are observed as well. Furthermore, the optical fractal states increase exponentially with the increase of the sequence number. The number of the optical fractal states is obtained by the theoretical calculation and the numerical simulation. The results show that it is matched very well between the two simulating methods. Moreover, the optical fractal effect depends on the incident angle of light, of which the optical fractal states can split/incorporate by modulating the incident angle of light. The optical fractal states incorporate as the incident angle of light changes. Wide passbands appear at the incident angle of light *θ*_*i*_ = 65°. If one keeps increasing of the incident angle of light, the splitting of the optical fractal states appears. However, the splitting cannot recovers the optical fractal states into the beginning states and some new optical fractal states are formed. This study could be utilized for band-pass filters and reflectors.

## Supporting information

S1 Dataset (RAR)
